# Robust Retinal Blood Vessel Segmentation Based on Reinforcement Local Descriptions

**DOI:** 10.1155/2017/2028946

**Published:** 2017-01-18

**Authors:** Meng Li, Zhenshen Ma, Chao Liu, Guang Zhang, Zhe Han

**Affiliations:** Qianfoshan Hospital of Shandong Province, Jinan 250014, China

## Abstract

Retinal blood vessels segmentation plays an important role for retinal image analysis. In this paper, we propose robust retinal blood vessel segmentation method based on reinforcement local descriptions. A novel line set based feature is firstly developed to capture local shape information of vessels by employing the length prior of vessels, which is robust to intensity variety. After that, local intensity feature is calculated for each pixel, and then morphological gradient feature is extracted for enhancing the local edge of smaller vessel. At last, line set based feature, local intensity feature, and morphological gradient feature are combined to obtain the reinforcement local descriptions. Compared with existing local descriptions, proposed reinforcement local description contains more local information of local shape, intensity, and edge of vessels, which is more robust. After feature extraction, SVM is trained for blood vessel segmentation. In addition, we also develop a postprocessing method based on morphological reconstruction to connect some discontinuous vessels and further obtain more accurate segmentation result. Experimental results on two public databases (DRIVE and STARE) demonstrate that proposed reinforcement local descriptions outperform the state-of-the-art method.

## 1. Introduction

Retinal fundus images play an important role for diagnose and treatment of cardiovascular and ophthalmologic diseases [1–3]. However, the manual analysis of the retinal fundus image is time-consuming and needs the empirical knowledge. Therefore, it is necessary for developing automatic analysis of retinal fundus images. Retinal blood vessel segmentation is the fundamental work of retinal fundus images analysis because some attributes of retinal blood vessels [4], such as width, tortuosity, and branching pattern, are important symptoms of diseases. Besides, retinal blood vessels segmentation is also useful for other applications such as optic disk detection. Based on the position of vessels, optic disk and fovea in the funds image can be detected through their relative location to blood vessels [5].

Some methods are proposed for retinal blood segmentation. The methods can be divided into two categories: supervised methods and unsupervised methods. Supervised methods obtain segmentation results with labeled images whereas unsupervised methods do not need labeled image.

Supervised methods obtain segmentation results with labeled images whereas unsupervised methods do not need labeled image. Unsupervised methods mainly contain four categories: matched filtering, vessel tracking, morphology processing, and model based algorithms. Matched filtering methods generally apply filters such as Gaussian filters or its variation to fit the shape and gray distributions of vessels and use the corresponding responses to detect vessels. Tracking algorithm based on the connectivity of vessels [6] is proposed for tracking the vessel by starting from an initial vessel point and finding next point belonging to vessel according to some designed rules. Morphology processing based method is usually combined with other properties of vessels, to get vessel-like structures from retinal images [7–9]. Model based methods mainly contains Hermite model [10], vector fields [11], level set [12, 13], region growing [13], active contour [14], and other methods [9].

The supervised segmentation methods firstly regard each pixel as an instance and extract feature of them. Then, training instances are selected for training segmentation model. For a test image, the image is segmented by the trained segmentation model, which is giving the label of the pixels. For these methods, feature extraction and segmentation model construction are the two key factors; some related feature and classifiers are proposed such as image ridge based features and KNN [15], 2D Gabor wavelet and GMM classifier [16], line operators and support vector classification [17], virtual template expansion based features and cellular neural networks [18], integrated features and AdaBoost classifier [19], gray-level and moment invariants-based features and feed-forward neural network [20], and pathological and vessel structure considering features and boosted decision trees [21]. These features are designed manually while [22, 23] employ deep learning for learning the feature automatically. Although deep learning can achieve the better performance, the parameters tune is complicated. Line operator based features are also proposed to capture the local shape information of vessels [17, 24, 25].

**Figure 1:**
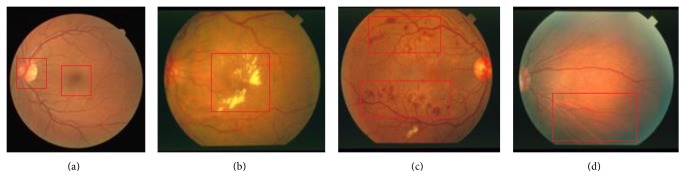
The edges of other structures in retinal image and pigmented epitheliums in the background are shown in red boxes. (a) OD and fovea (test_01 in DRIVE database); (b) bright lesions in pathological image (im0001 in STARE database); (c) dark lesions in pathological image (im0139 in STARE database); (d) pigmented epitheliums (im0291 in STARE database).

Generally, the performance of supervised segmentation methods has more effect than unsupervised segmentation methods due to full use of the label information. However, conventional features used in supervised segmentation method only reflect one aspect of the retinal blood vessels such as shape, size, or connectivity of blood vessels. Insufficient information may be sensitive to other structures in retinal image (as shown in Figure 1) such as OD and bright and dark lesions in pathological image [26], resulting in segmentation performance degradation. In addition, some discontinuous vessels after segmentation may appear due to the influence of noise or illumination.

To solve these two problems, we propose retinal vessel segmentation method based on reinforcement local descriptions. We firstly proposed the line sets based feature by employing the length prior of vessels, which capture the local shape information of vessels. Then we develop the ensemble features by fusing the line sets based feature and local intensity feature with morphology gradient feature. General local features only reflect one characteristic of vessels. For example, line set based features only reflect local shape of vessels. However, the proposed ensemble features can capture local shape, local intensity, and local edge information of vessels, which can reinforce description of local characteristics. Therefore, we refer to the proposed ensemble features as reinforcement local description in this paper. Moreover, the postprocessing method based on morphological reconstruction is also proposed to deal with the problem of the discontinuous vessels and further improve the segmentation result. We summarize the contribution of this paper as follows:

1. A novel line set based feature is proposed to capture local shape information of vessels by employing the length prior of vessels, which is robust to intensity variety.
2. In order to improve the performance of the features, the proposed line set based feature, local intensity, and morphological gradient feature are combined to generate reinforcement local descriptions which contain more effective local information of vessels.
3. Morphological reconstruction is employed to connect some discontinuous vessels to further improve segmentation result.

To be noticed, this paper is the extension of [26], compared with previous work of [26]. We improve the local shape feature by expanding our line set from one to two line sets, which can capture more useful information of the tiny vessels; in addition, we also develop postprocessing method to connect the discontinuous thin vessels. The experiments are also supplemented to demonstrate the effectiveness of the proposed method.

## 2. Materials and Methods

### 2.1. Data Description

We evaluate the performance of proposed method on two public databases. One is STARE (Structured Analysis of the Retina) which is collected by Hoover et al. [27]. In this database, 20 raw retinal images are used for blood vessel segmentation and ten of them have pathologies. All images were captured by a TopCon TRV-50 fundus camera at 35° field of view (FOV) and digitized to 700 × 605 pixels with 8 bits per color channel. The FOV for each image is approximately 650 × 550 pixels. There were two ground-truth image sets labeled by two observers. In the two sets, 10.4% and 14.9% of pixels are labeled as vessels, respectively. We use the first result as ground truth. The other database is DRIVE which was established by Alonso-Montes et al. [28]. In the database, 20 images are employed for training and 20 images for testing. These images are captured by a Canon CR5 3CCD camera with a 45° FOV and size is 700 × 605 pixels per color channel and has a FOV of approximately 540 pixels in diameter. Set A and set B, respectively, marked 12.7% and 12.3% pixels as vessels by two observers. Performance will be evaluated on set A.

In order to qualify the performance of our method, we compare our results with the gold standard, which is segmented by medical experts. All the metrics are evaluated according to the numbers of pixels in FOV. The performance evaluation metrics are shown in Table 1.

In Table 1, TP denote the vessel pixel which are segmented as vessel, and the FP denote the background pixels which are segmented as vessel incorrectly. TN denote the background pixels which are segmented as background, and the FN denote the vessel pixels which are segmented as background incorrectly. Accuracy (ACC) is the ratio of all the correctly classified pixels in all pixels of FOV. Sensitivity (SEN) is the ratio of correctly classified pixels in all the vessel pixels. Specificity (SPE) is the percentage of correctly classified background pixels in all the background pixels.

### 2.2. Methods

**Figure 2:**
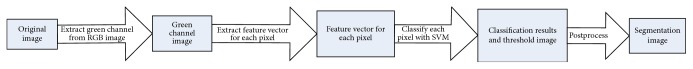
Flowchart of the proposed method.

The proposed method is presented in this section. Green channel is firstly extracted from the original RGB image due to its higher contrast. And then the line sets based feature, local intensity, and multiscale morphology feature are extracted and combined into the reinforcement local descriptions for each pixel. After feature extraction, SVM is trained based on the reinforcement local descriptions and used for vessel segmentation. Finally, postprocessing is proposed for obtaining more accurate segmentation image. Flowchart of our method is shown in Figure 2.

#### 2.2.1. Reinforcement Local Description

*Line Set Based Feature*. The shape of blood vessel can be seen as rectangle due to its line segments structure [29]. Therefore, the local blood vessels can be regarded as a local rectangle area approximately. To represent the local rectangle characteristics of blood vessel, we propose the line sets based features. The line sets are used for searching the blood vessels pixels on local rectangle.

**Figure 3:**
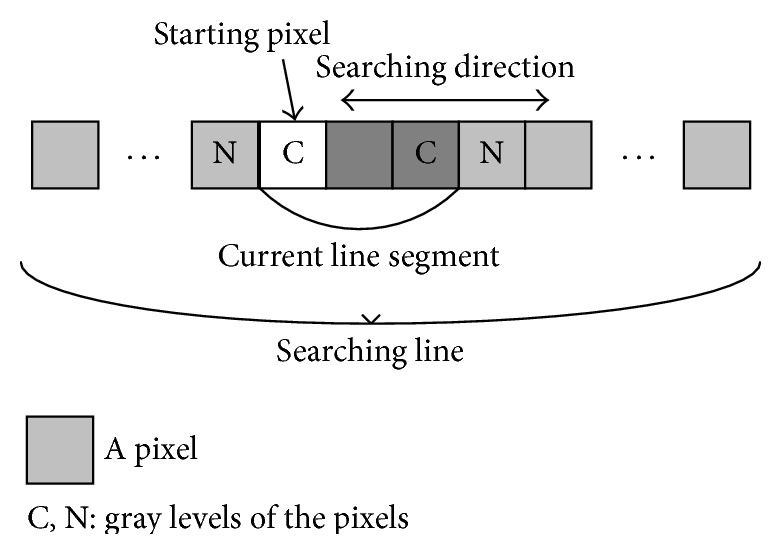
Explanation of searching step.

**Figure 4:**
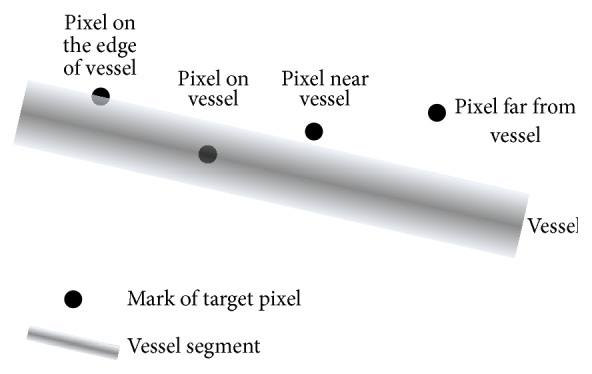
Four categories of pixels in retinal images. Pixels in retinal images are roughly classified into four categories: pixel on the edge of vessel, pixel on vessel, pixel near vessel, and pixel far from vessel.

**Figure 5:**
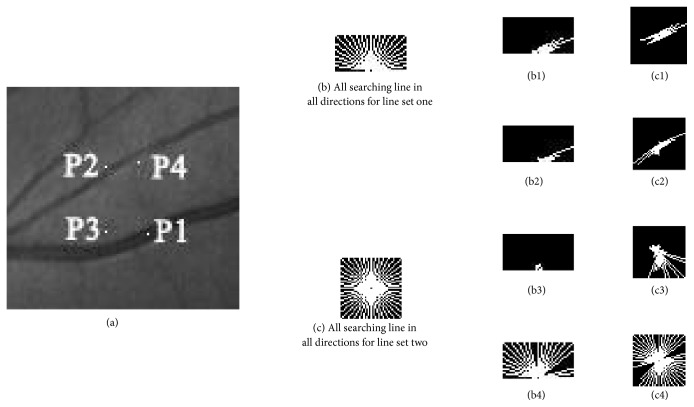
Examples of line sets. We select four kinds of points: on the central of vessel (P1), on the edges of vessel (P2), above the vessel (P3), and under the vessel (P4) in green channel image (a). All searching lines in all directions for line set one and line set two are shown in (b) and (c). Searching step is starting from the central pixel and along these lines in all direction. The corresponding line sets of each pixel in local area are shown. (b1), (b2), (b3), and (b4) are the line sets one of P1, P2, P3, and P4. (c1), (c2), (c3), and (c4) are the line sets two for P1, P2, P3, and P4.

Line sets based features for each pixel are designed to represent the shape characteristics of a local area which contains the pixel. Firstly, two line sets are extracted for a pixel by searching step. A line set contains many line segments obtained by searching step. Line segments are firstly extracted in many directions in order to represent the shape of local area; then features are extracted based on all the line segments. In the searching step, the searching line is starting from the instance pixel and along a certain direction. Initially, only the staring pixel is in current search line. In this paper, if the intensity difference among the pixel and neighbor pixels is less than* S*; this pixel on searching direction satisfies searching condition. The pixel which satisfied this condition will be added to current line. Otherwise, stop searching. While stopping the search a line segment is obtained. Twenty-four lines segment is obtained in every 7.5 degrees, which is enough to fill a local area, for line set one and line set two. The searching lines are numbered from 1 to 24 starting from *x*-axis and 24 line segments are enough to cover the local area. The length of each searching line for line set one is 21 because 21 pixels is close to the biggest width of blood vessels, while length of searching line for line set two is 41 for the reason that 41 pixels is certainly more than the width of blood vessels so that the shape of blood vessels can be better represented. The searching condition is described in Figure 3. In ideal condition, all line segments for the pixels on vessel would end with the edges of vessels, and the line segments for the pixels in background would end with the end of searching line, noise points, or edges of vessels. Furthermore, pixels in retinal images are roughly classified into four categories: pixels on the edge of vessel, pixels on vessel, pixels near vessel, and pixels far from vessel. The four categories and the corresponding line sets are also shown in Figures 4 and 5.

Then, line sets based feature are extracted based on the two line sets. The longest line segment in line set is denoted as L and the shortest of line segment is denoted as P. The length of L and P is denoted as LL and LP, respectively. The lengths sum of all line segments in a line set is denoted as AL. The values of LL, LP, LL-LP, LL-AL/24, and LP-AL/24 are ability to roughly present the size and shape of local area. The direction number of L is defined as main direction number. The ratio of the number of pixels, which have same main direction number with the starting pixel on a line segment, to the length of the line segment is denoted as R. For each pixel, the sum of LL and LP in eight-neighborhood is denoted as SNLL and SNLP, respectively.

The line set based feature can represent the characteristics of the local rectangle, which can distinguish between vessel and background or noise. The values of RL + RP, *‖*SNLL/8-LL*‖*, and *‖*SNLP-LP*‖* are calculated as the features to capture the local information of a pixel. Examples of these features are show in Table 2.

In Table 2, P1, P2, P3, and P4 are the four points in Figure 5(a). In this table, f1, f2, f3, f4, f5, f6, f7, and f8 denote LL, LP, LL-LP, LA-AL/24, AL/24-LB, RL + RP, *‖*SNLL/8-LL*‖*, and *‖*SNLP-LP*‖* for a line set. We extract all the features for the four points. From the table, we can see that, for pixels P1 and P2, f1 is high and f2 is relatively low; therefore f3 and f4 are high and f5 is low. Besides, for background pixels P3 and P4, f1, f2, f3, f4, and f5 are irregular. For pixels P2 and P4, f6 and f7 are low and f8 is high. We can see that pixels far from edges have restively higher f6 and f7 and lower f8.

Compared with existing line operator feature [17] extracted according to intensity difference, the proposed line set feature is extracted by employing the length prior of vessels, which is more robust to intensity variety.

*Local Intensity Feature*. We also extract local intensity feature from the green channel image because blood vessels are darker than background. For each pixel **P**, we obtain the square area whose size is *L* × *L* and center is **P**. We calculate the mean intensity of this area as the local intensity feature of pixel **P**. The mean intensity feature is more robust than intensity of pixel **P**. In addition, considering different size of the vessel, we calculate the local intensity feature based on different size of square area. In our experiment, we set the value of **L** as 3, 5, 11, 21, 31, and 41 for capturing the intensity characteristics of the vessel pixels more accurately.

*Morphological Gradient Feature*. Because of the noise or illumination, some local edge of the small vessels may be segmented incorrectly, to make the feature more robust; morphological gradient is employed to enhance the local edge of tiny vessels.

Morphological gradient is the difference between the dilation and the erosion of an image and can be used for enhancing the edges of bright region which can be regarded as the blood vessels on certain degree. We denote *f* ⊕ *d*_*i*_ and *f* ⊗ *d*_*i*_ as dilation operation and erosion operation for image *I* with structural element *d*_*i*_. *d*_*i*_ denote the disk structure with radius *i*. Because of the different widths of blood vessels, mean value of multiscale morphological gradient feature is calculated as in the following equation:

(1) $$\begin{array}{c} F\_grad=\frac{1}{6}\overset{6}{\underset{i=1}{\sum }}\left(\;I⊕d_{i}-I⊗d_{i}\;\right)\;\text{s.t }i=1,2,3,4,5,6. \end{array}$$

The reinforcement local descriptions are generated by combining the line set based feature, local intensity feature, and morphological gradient feature into a vector. The descriptions are normalized to avoid large differences of feature values between different retina images.

#### 2.2.2. Segmentation

We use SVM as the segmentation model due to the perfect theory foundation [30–32] and its better generation ability [33]. We firstly choose some images as the training image, and the blood vessel segmentation result of these images can be obtained by the manual label. And then, we select the pixels from the training images as the training data. We extract the reinforcement local descriptions of each training pixel and label these pixels as blood vessel or background according to the manually labeled segmentation result image. Finally, these training pixels are used for training SVM. In the experiment, RBF kernel is used for SVM. The parameters *C* and the bandwidth for RBF kernel are selected through 5-fold cross-validation on the training data. Based on the training data, parameters *C* and bandwidth for RBF kernel are obtained and the values of them are 1 and 0.01, respectively.

#### 2.2.3. Postprocessing

After vessel segmentation by SVM, some discontinues thin vessels exist. To connect the discontinuous vessels and obtain more accurate segmentation result, we propose the postprocessing based on the morphological reconstruction.

**Figure 6:**
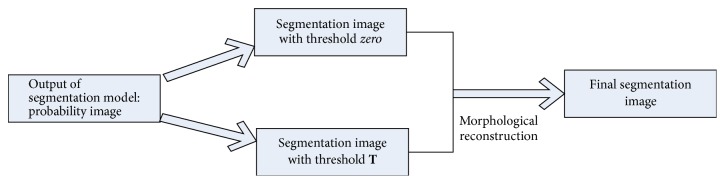
Flowchart of postprocessing.

The output of support vector classifier is a probability image. The probability represents the likelihood of the pixel belong to the blood vessels. Based on the probability image, a threshold zero is given to separate blood vessels and background. In these segmented images, some noise may exist. In addition, another binary image is obtained by giving a higher threshold **T**, and the obtained image can remove some noise. However, higher threshold can make some thin vessels discontinuous. To connect the discontinuous thin vessels, we are using the morphological reconstruction on the segmented image obtained via threshold **T**, and the template is the binary image obtained via threshold zero (as shown in Figure 6). The morphological reconstruction can connect the discontinuous vessels which are connected on the binary image obtained via threshold zero without introducing the noise. Therefore, the postprocessing can achieve more accurate segmentation result. In experiment, the values of threshold **T** are set as 0.2 on STARE and 0.35 on DRIVE.

## 3. Results and Discussion

### 3.1. Analysis of Parameter

In classification step, training set is constructed for training SVM. For DRIVE database, 20000 pixel samples are randomly selected from each training image for constructing the training set. The same pixel sample selection method is used for DRIVE database. Leave-one-out strategy is used for testing. By using leave-one-out strategy, for each image, this image is testing image and the left images are training images, and the mean performance is the final performance.

**Figure 7:**
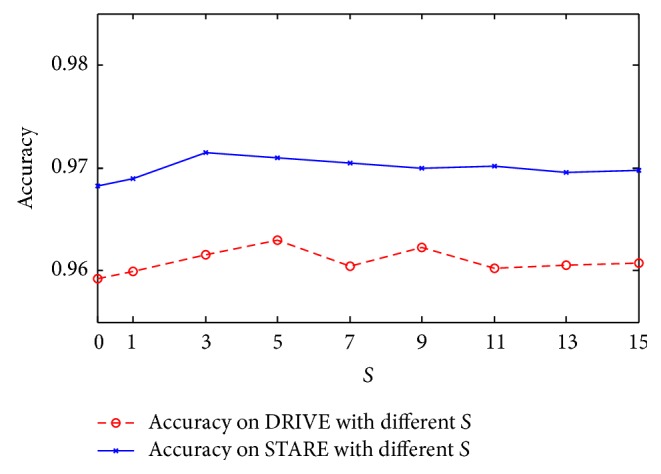
The accuracy with different values of *S* on STARE and DRIVE.

We analyze the influence of parameter **S** in the feature based line set extraction step. The accuracy with different *S* on STARE and DRIVE is shown in Figure 7. From this figure, we can see that the best results are achieved on both databases when the value of **S** is 5. Moreover, the accuracy with variation of *S* is stable; therefore, the segmentation method is less sensitive to the setting of parameter *S*. In the next experiments of this paper, value of parameter **S** is set as 5.

### 3.2. Analysis of Postprocessing

**Figure 8:**
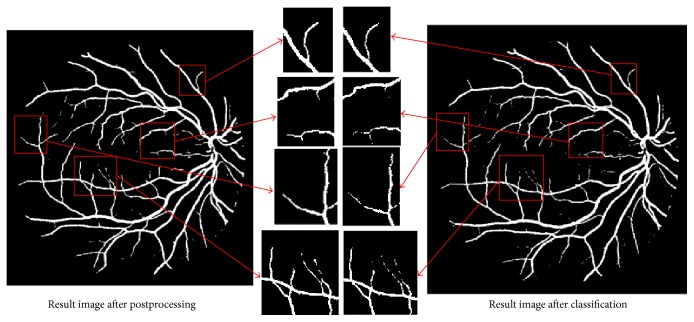
Comparison between result image after postprocessing and result image only after classification.

After vessel segmentation by SVM, some discontinues thin vessels appeared due to some noise. The absence of thin vessels causes the lower sensitivity. However, postprocessing based on morphological reconstruction can enhance the connectivity of thin vessels on certain degrees (as shown in Figure 8), which can improve the segmentation result.

### 3.3. Comparison with the State-of-the-Art Methods

The experiment results compared with the state-of-the-art methods on DRIVE and STARE databases are shown in Tables 3 and 4. All the methods are listed by year. The 2nd human observer means the performance of the segmentation results given by the other expert.

From Tables 3 and 4 we can see that the performance of the proposed method is the best. The state-of–the-art methods can reflect only one characteristic of vessels. For example, [17] only captures local shape information of vessels. However, proposed reinforcement local description contains the more local information of the shape and gray and enhanced edge, which is more robust and effective. In addition, the postprocessing can connect some discontinuous vessels and further made the segmentation result more accurate.

### 3.4. Performance in Pathological Images

Performance comparison of results on pathological retinal images is shown in Table 5. From Table 5 we can see that the proposed method is also robust to the pathological images. The reason may be that morphological gradient feature in the reinforcement local description can enhance the edge, and the shape and gray information made the feature less sensitive to the bright and dark lesions. Therefore, reinforcement local description is more robust to the variation of the blood vessels in pathological retinal images. In addition, the postprocess can abandon some noise and connect some discontinuous vessels, which made our segmentation result more accurate.

### 3.5. Performance of Cross Training

Furthermore, the performance of the proposed method is stable relatively when the data set and some parameters are variants. We randomly select 1000 pixel samples from each image on DRIVE as the training set and test on STARE; the accuracy is 0.9626 as shown in Table 6, while training images are from STARE and testing images from DRIVE; the accuracy is 0.9531 as shown in Table 6. From the experiment result, we can see that ACC is stable relatively when the training set varied, which is important for clinical application.

## 4. Discussion

In this paper, we propose a robust vessel segmentation method based on the reinforcement local description. The reinforcement local descriptions contain line set based feature, local intensity feature, and morphology gradient feature. The line set based features can represent the local shape of the vessels, local intensity feature can reveal the gray information of the local area, and the morphology gradient feature can enhance local edge of small vessels, which made the reinforcement local description more robust. Moreover, the postprocess based on morphological reconstruction can connect the discontinuous vessels.

From the experiment result, we can see that our method can achieve the best performance due to the use of more useful information and the postprocessing. The high specificity can avoid introducing the noise. Sensitivity is high on STARE database and is accessible on DRIVE database. Besides, with the high accuracy on both databases, our method can achieve a satisfactory segmentation results. For pathological image, the proposed method also outperforms other methods due to the rich useful information of the reinforcement local description. The postprocessing can also enhance the connectivity of thin vessels, which can improve the sensitivity. The proposed method also achieves better experiment result on cross training due to the robustness of the reinforcement local description. Above all, the stable property our algorithm is important for clinical application.

## 5. Conclusion

In this paper, retinal blood vessel segmentation method based on the reinforcement local description is proposed. For each pixel, line sets based feature is firstly developed for representing the shape of the blood vessel. The proposed line set feature is extracted by employing the length prior of vessels, which is more robust to intensity variety. And then, line sets based feature, local intensity feature, and morphology gradient feature are combined for obtaining more effective reinforcement local descriptions. The descriptions contain the rich local information of the shape and gray and enhanced edge, which is more robust. After feature extraction, SVM is trained for vessel segmentation. Finally, postprocessing is proposed for further obtaining more accurate segmentation result. The experiment results on DRIVE database and STARE database demonstrate the effectiveness of the proposed method. Future work will be focused on the designing of more effective segmentation model. Ensemble learning may be a way for boosting the performance of the classifiers.

## Figures and Tables

**Table 1:** All the metrics for performance evaluation.

Measure	Description
Accuracy (ACC)	(TP + TN)/FOV pixel count
Sensitivity (SEN)	TP/(TP + FN)
Specificity (SPE)	TN/(TN + FP)

**Table 2:** Examples of features.

	Features based on line set one								Features based on line set two							
	f1	f2	f3	f4	f5	f6	f7	f8	f1	f2	f3	f4	f5	f6	f7	f8
P1	21	2	19	15.21	3.80	0.15	81	7	31	4	27	20.54	6.46	0.30	140	8
P2	21	3	18	16.58	1.42	0.50	15	45	22	4	18	12.17	5.83	0.81	73	100
P3	9	2	7	3.38	3.63	0	45	32	29	6	23	11.54	11.46	0.071	8	57
P4	21	21	0	0	0	1.35	0	28	41	41	0	2.38	−2.38	0.33	0	71

**Table 3:** Segmentation results of different methods on DRIVE database.

Type	Methods	Year	SEN	SPE	ACC
Supervised methods	2nd human observer	—	0.7796	0.9717	0.9470
	Staal et al. [15]	2004	N.A	N.A	0.9442
	Soares et al. [16]	2006	N.A	N.A	0.9466
	Ricci and Perfetti [17]	2007	N.A	N.A	0.9563
	Welikala et al. [24]	2014	0.737	0.9521	0.942
	Nguyen et al. [25]	2013	0.7321	0.9487	0.9407
	Lupascu et al. [19]	2010	0.7200	N.A	0.9597
	Marín et al. [20]	2011	0.7067	0.9801	0.9452
	You et al. [8]	2011	0.7410	0.9751	0.9434
	Fraz et al. [21]	2012	0.7406	0.9807	0.9480
	Li et al. [22]	2016	0.7569	0.9816	0.9527
	Proposed method	2016	**0.7680**	**0.9827**	**0.9630**

Unsupervised methods	Mendonça and Campilho [34]	2006	0.7344	0.9764	0.9452
	Alonso-Montes et al. [28]	2008	N.A	N.A	0.9185
	Lam et al. [9]	2010	N.A	N.A	0.9472
	Delibasis et al. [35]	2010	N.A	N.A	0.9407
	Qian Zhao et al. [13]	2014	0.7354	0.9789	0.9477

**Table 4:** Segmentation results of different methods on STARE database.

Type	Methods	Year	SEN	SPE	ACC
Supervised methods	2nd human observer	—	0.8951	0.9384	0.9348
	Staal et al. [15]	2004	N.A	N.A	0.9516
	Soares et al. [16]	2006	N.A	N.A	0.9480
	Ricci and Perfetti [17]	2007	N.A	N.A	0.9584
	Welikala et al. [24]	2014	0.7448	0.9533	0.9431
	Nguyen et al. [25]	2013	0.7314	0.9429	0.9324
	Marín et al. [20]	2011	0.6944	0.9819	0.9526
	You et al. [8]	2011	0.7260	0.9756	0.9497
	Fraz et al. [21]	2012	0.7548	0.9763	0.9534
	Li et al. [22]	2016	0.7726	0.9844	0.9628
	Proposed method	2016	**0.7890**	**0.9883**	**0.9710**

Unsupervised methods	Hoover et al. [27]	2000	0.6747	0.9565	0.9264
	Mendonça and Campilho [34]	2006	0.6996	0.9730	0.9440
	Lam and Yan [11]	2008	N.A	N.A	0.9474
	Lam et al. [9]	2010	N.A	N.A	0.9567
	Delibasis et al. [35]	2010	N.A	N.A	0.9324
	Qian Zhao et al. [13]	2014	0.7187	0.9767	0.9509

**Table 5:** Performance comparison of results on pathological retinal images on STARE database.

Methods	SEN	SPE	ACC
2nd human observer	0.8719	0.9384	0.9324
Hoover et al. [27]	0.6587	0.9565	0.9258
Soares et al. [16]	0.7181	0.9765	0.9500
Fraz et al. [21]	0.7262	0.9764	0.9511
Li et al. [22]	0.78	0.98	0.9672
Proposed method	**0.7843**	**0.9837**	**0.9690**

**Table 6:** ACC comparison of different methods with cross training.

Method	DRIVE (trained on STARE)	STARE (trained on DRIVE)
Soares et al. [16]	0.9397	0.9327
Ricci and Perfetti [17]	0.9266	0.9464
Marín et al. [20]	0.9448	0.9528
Fraz et al. [21]	0.9456	0.9493
Li et al. [22]	0.9486	0.9545
*Proposed method*	**0.9531**	**0.9626**
